# Skeletal coccidioidomycosis: an atypical pediatric presentation

**DOI:** 10.1007/s00256-026-05313-0

**Published:** 2026-07-28

**Authors:** Lane H. McCoy, John S. A. Chrisinger, Benjamin E. Northrup, Jonathan C. Baker

**Affiliations:** 1 Washington University School of Medicine, 660 S Euclid Ave, St Louis, MO USA; 2 Department of Pathology and Immunology, Washington University School of Medicine, 660 S Euclid Ave, St Louis, MO USA; 3 Mallinckrodt Institute of Radiology, Washington University School of Medicine, 510 S Kingshighway Blvd, St Louis, MO USA

**Keywords:** Osteomyelitis, Skeletal coccidioidomycosis, Magnetic resonance imaging

## Abstract

Coccidioidomycosis is most commonly a self-limited pulmonary infection. Disseminated disease is rare, occurring in fewer than 1% of cases, with skeletal involvement representing an uncommon manifestation in children. We report a case of skeletal coccidioidomycosis in a previously healthy 12-year-old with pelvic involvement and no antecedent pulmonary symptoms. The patient presented with 1.5 months of progressive, atraumatic left pelvic pain, night sweats, and weight loss, without respiratory complaints. Radiographs demonstrated a lytic lesion of the left ilium extending toward the acetabulum, with ill-defined margins and prominent surrounding reactive sclerosis. Mineralized matrix production was absent. Magnetic resonance imaging revealed an extensive marrow-replacing lesion contiguous with a heterogeneous soft-tissue component exhibiting focal internal enhancement. The lesion demonstrated diffusion restriction, consistent with abscess formation, while the hip joint was preserved and there was no neurovascular involvement. Although these findings raised concern for malignancy, there were subtle infection-favoring features and subsequent biopsy was performed. Histopathology demonstrated fungal elements with associated giant cell reaction. The patient was treated initially with intravenous amphotericin B and transitioned to oral itraconazole following culture confirmation of Coccidioides, with a planned prolonged antifungal course. At 6-month follow-up, the patient had resumed full activity with complete resolution of pain and preserved pelvic function. This case highlights the importance of including coccidioidomycosis in the differential diagnosis of aggressive pediatric osseous lesions. Recognition of key imaging features, including lytic osseous destruction with surrounding sclerosis and a heterogeneous soft-tissue component with diffusion restriction, can facilitate timely diagnosis, prevent misdiagnosis, and reduce morbidity risk.

## Introduction

Coccidioidomycosis, an endemic fungal infection caused by *Coccidioides* species, typically presents as a self-limited pulmonary illness. Its geographic distribution spans the arid regions of the southwestern United States, northern Mexico, and parts of Central and South America, with sporadic cases reported in travelers from these areas. The geographic range of reported cases has expanded in the past few years, with emerging cases in previously nonendemic regions. Recent epidemiologic data suggest that incidence has also risen in endemic regions, likely driven by climate variability affecting soil ecology, population growth, and increased exposure to airborne arthroconidia through the environment or human soil disruption [1–3]. Disseminated disease occurs in fewer than 1% of cases, with skeletal involvement representing a particularly serious but uncommon manifestation [4,5]. Pediatric skeletal coccidioidomycosis is rare, and when present frequently mimics bacterial osteomyelitis or primary bone malignancy, leading to delayed diagnosis and suboptimal management [6–8]. Limited data exist describing the epidemiology, presentation, and outcomes of pediatric disseminated coccidioidomycosis resulting in osteomyelitis.

Imaging evaluation of skeletal coccidioidomycosis can be challenging. Radiographs may reveal focal lytic or articular lesions, often with aggressive periosteal reaction, while magnetic resonance imaging (MRI) frequently demonstrates marrow-replacing lesions with contiguous heterogeneous soft-tissue components. General radiographic features such as rim-enhancing fluid collections, restricted diffusion, or T1 “penumbra” signals can favor infection, whereas mineralized matrix production, aggressive periosteal reaction, and absence of soft-tissue abscess suggest neoplasm. The combination of osseous destruction with surrounding sclerosis may further suggest a chronic infectious process, although these findings are not specific. Despite these subtleties, overlap with bacterial or mycobacterial osteomyelitis and primary bone tumors often necessitates biopsy and microbiologic confirmation for definitive diagnosis [9–13].

Skeletal involvement carries significant morbidity; untreated or mismanaged disease can result in progressive bone destruction, joint dysfunction, and long-term disability [5,14]. Early recognition and targeted antifungal therapy are critical, particularly in pediatric patients where delayed intervention may affect skeletal growth and function. Case reports and series highlight the potential for limb-sparing outcomes when appropriate antifungal therapy is instituted promptly [5,12].

We describe a case of skeletal coccidioidomycosis in a previously healthy 12-year-old boy with isolated pelvic involvement and no preceding pulmonary symptoms. This case is notable for isolated osseous involvement without clinically recognized antecedent pulmonary symptoms and the diagnostic value of sequence-specific MRI findings prompting biopsy and early antifungal therapy. This case report also emphasizes the importance of considering atypical fungal infection in pediatric osseous lesions in endemic regions or patients with relevant travel history, to highlight imaging features that may favor infection over neoplasm and to reinforce the role of biopsy in guiding timely and appropriate management of fungal osteomyelitis.

## Case report

A previously healthy 12-year-old boy presented with 1.5 months of progressive, atraumatic left pelvic pain. The patient reported associated night sweats and an unintentional 5-pound weight loss. There was no history of cough, dyspnea, fever, rash, or recent infections, and the patient was not immunocompromised. The patient resided in the midwestern United States and had no travel history including areas endemic for coccidioidomycosis. The patient had no known sick contacts, soil exposure, or activities associated with fungal spore inhalation. On physical examination, there was tenderness over the left iliac crest and lateral pelvis with mild limitation of hip flexion due to pain. There was no erythema, warmth, or swelling, and neurovascular examination was normal. No lymphadenopathy or organomegaly was present.

Laboratory studies demonstrated mild leukocytosis (WBC 14.2 × 10^3^/μL; normal: 4 × 10^3^/μL–10 × 10^3^/μL) with normal hemoglobin and platelet counts. ESR was 144 mm/hr (normal: < 15 mm/hr) and CRP 80.1 mg/L (normal: < 10 mg/L). Liver and renal function tests were normal. Blood cultures were negative. Interferon-gamma assay screening for tuberculosis and bacterial pathogen workup was unrevealing. Serologic testing was not obtained.

**Fig. 1:**
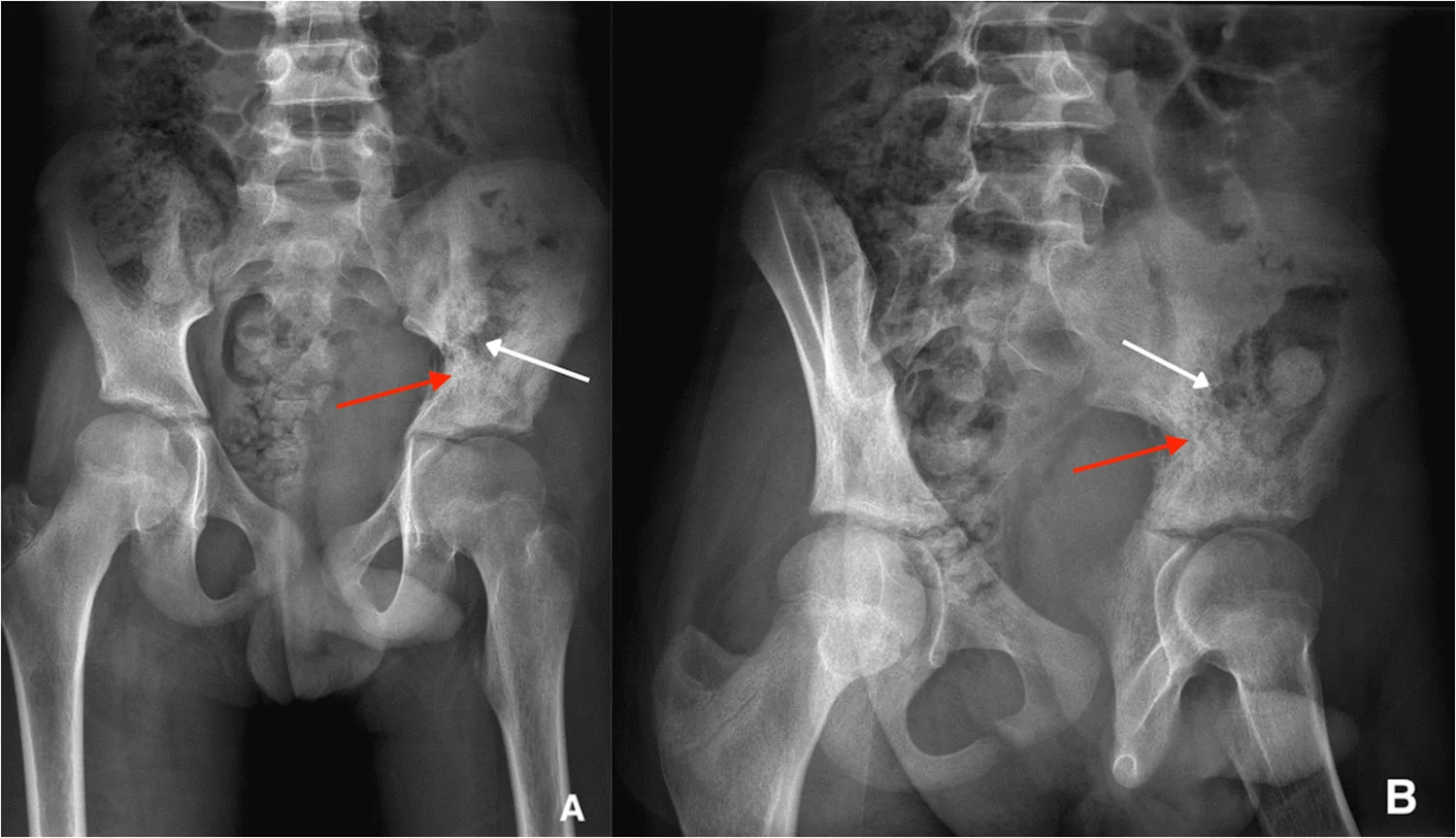
AP (**A**) and oblique (**B**) pelvis radiographs demonstrate an ill-defined osteolytic lesion in the left supra-acetabular ilium (white arrows) with prominent surrounding reactive sclerosis (red arrows) and a wide zone of transition. The combination of lysis and sclerosis suggests a relatively chronic or indolent osseous process despite its aggressive appearance Lesion conspicuity is somewhat limited by overlying bowel loops

**Fig. 2:**
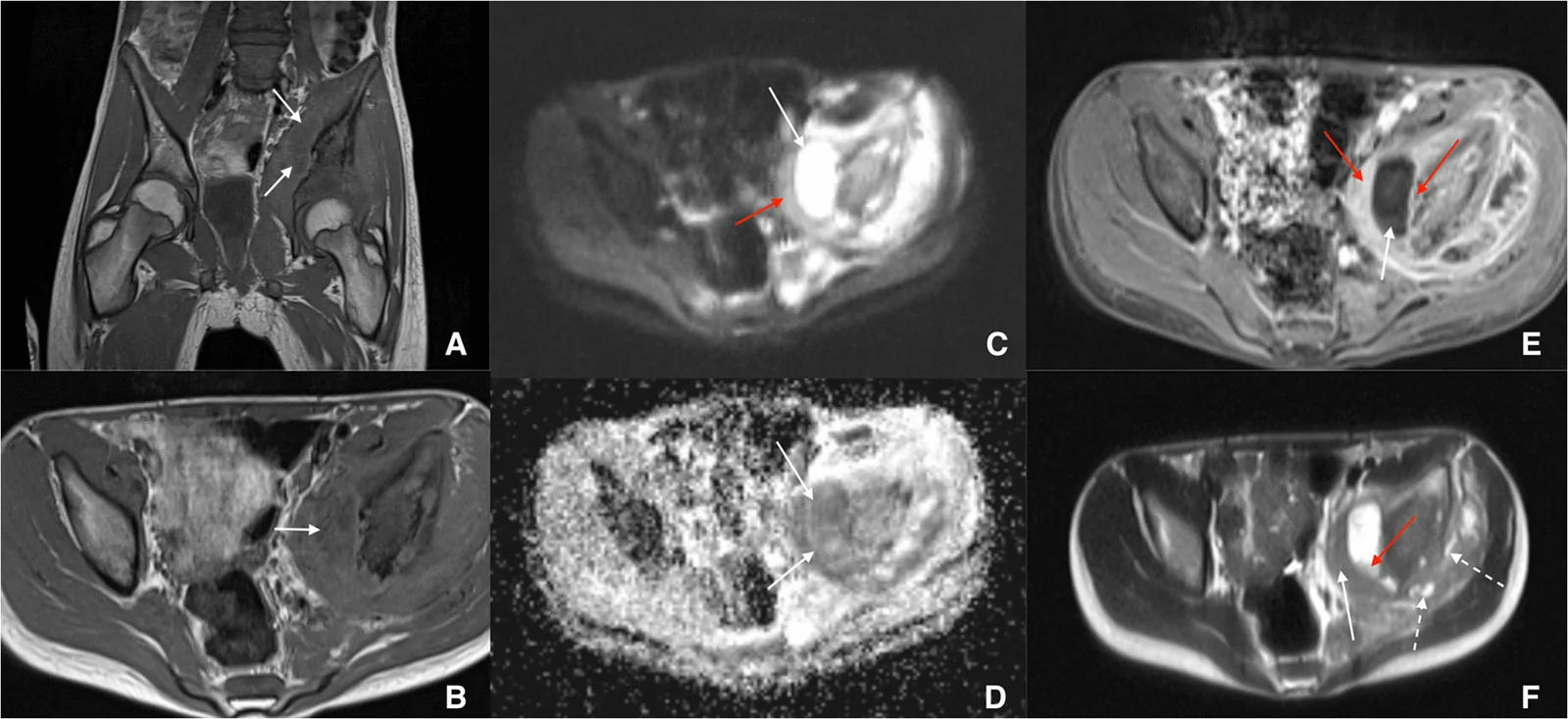
Coronal (**A**) and axial (**B**) T1-weighted MRI of the pelvis showed a hypointense soft-tissue and marrow-replacing lesion sparing the pelvic and triradiate cartilage (white arrows). Axial diffusion-weighted imaging suggested marked diffusion restriction (white arrow) within adjacent rim-enhancing soft-tissue collections (red arrow), consistent with abscess formation (**C**). ADC mapping confirms restricted diffusion with low-signal in the respective bone (white arrows) (**D**). Axial postcontrast T1 fat-suppressed images demonstrate medial and lateral rim-enhancing fluid collections (red arrows) adjacent to the lesion (white arrow), compatible with abscesses, with surrounding inflammatory enhancement (**E**). Axial T2 (**F**) imaging characterized extensive marrow, soft-tissue, and fascial edema surrounding the lesion (white arrow) with a focal fluid collection (red arrow) and smaller lateral abscesses (dashed arrows)

**Fig. 3:**
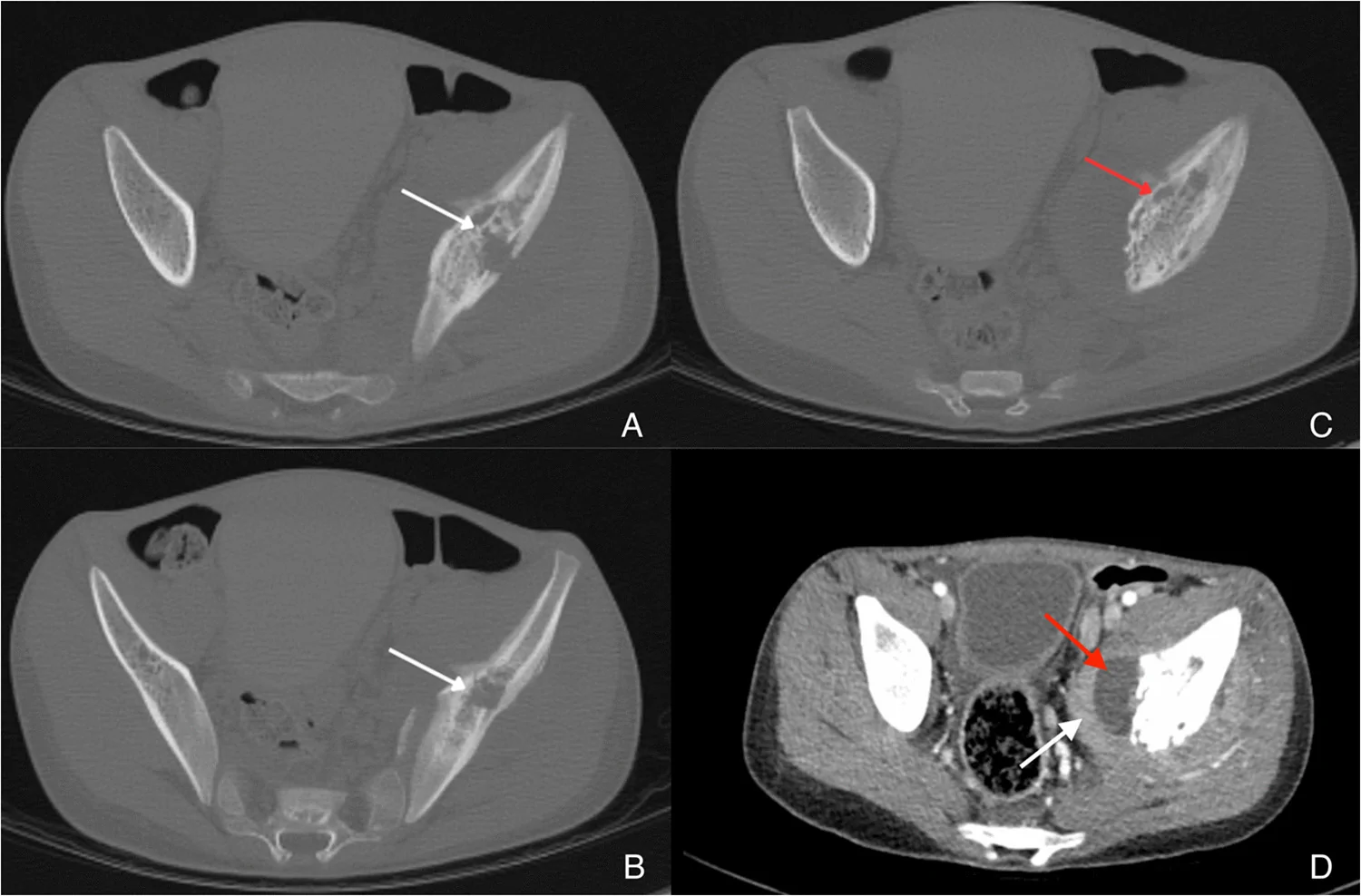
Axial computed tomography bone windows revealed cortical destruction (white arrows) (**A**, **B**) and the cloaking periosteal reaction (red arrow) (**C**) of the lesion. Soft tissue window visualized a hypodense fluid collection (red arrow) with obscuration of muscle planes representing edema (white arrow) (**D**)

Initial anteroposterior radiographs of the pelvis revealed a 4.5 × 3.0 cm ill-defined lytic lesion in the left supra-acetabular ilium with prominent surrounding sclerosis, extending toward the acetabulum (Fig. 1). There was no extension into the ischium or pubis. No osseous mineralized matrix was identified, and the wide zone of transition raised concern for aggressive pathology. The combination of lysis and surrounding sclerosis suggested a relatively chronic process despite the aggressive appearance. The acetabular articular surface remained intact with no joint space narrowing or widening. MRI demonstrated a marrow-replacing lesion measuring approximately 5.0 × 3.5 × 3.0 cm with areas of hypointense signal on T1-weighted images. On T2 sequences, the lesion was heterogeneously hyperintense with surrounding marrow and adjacent iliacus and gluteal muscle edema extending along adjacent fascial planes. Precontrast T1 fat-suppressed images showed low-signal areas within the soft tissue, while postcontrast T1 fat-suppressed images revealed multiple peripheral rim-enhancing collections within the medial and lateral soft tissues adjacent to the lesion compatible with abscess formation, with extensive surrounding inflammatory change (Fig. 2). Diffusion-weighted imaging demonstrated restricted diffusion within these rim-enhancing collections (ADC 0.8 × 10^−3^ mm^2^/s), supporting the presence of abscesses. The hip joint was preserved with no effusion or synovitis, and the growth plate was unaffected. There was no neurovascular encasement, sinus tracts, sequestra, or involucrum. Computed tomography better characterized the cortical disruption and cloaking periosteal reaction (Fig. 3). Chest radiograph and low-dose CT revealed no pulmonary involvement, and whole-body skeletal survey excluded multifocal disease.

**Fig. 4:**
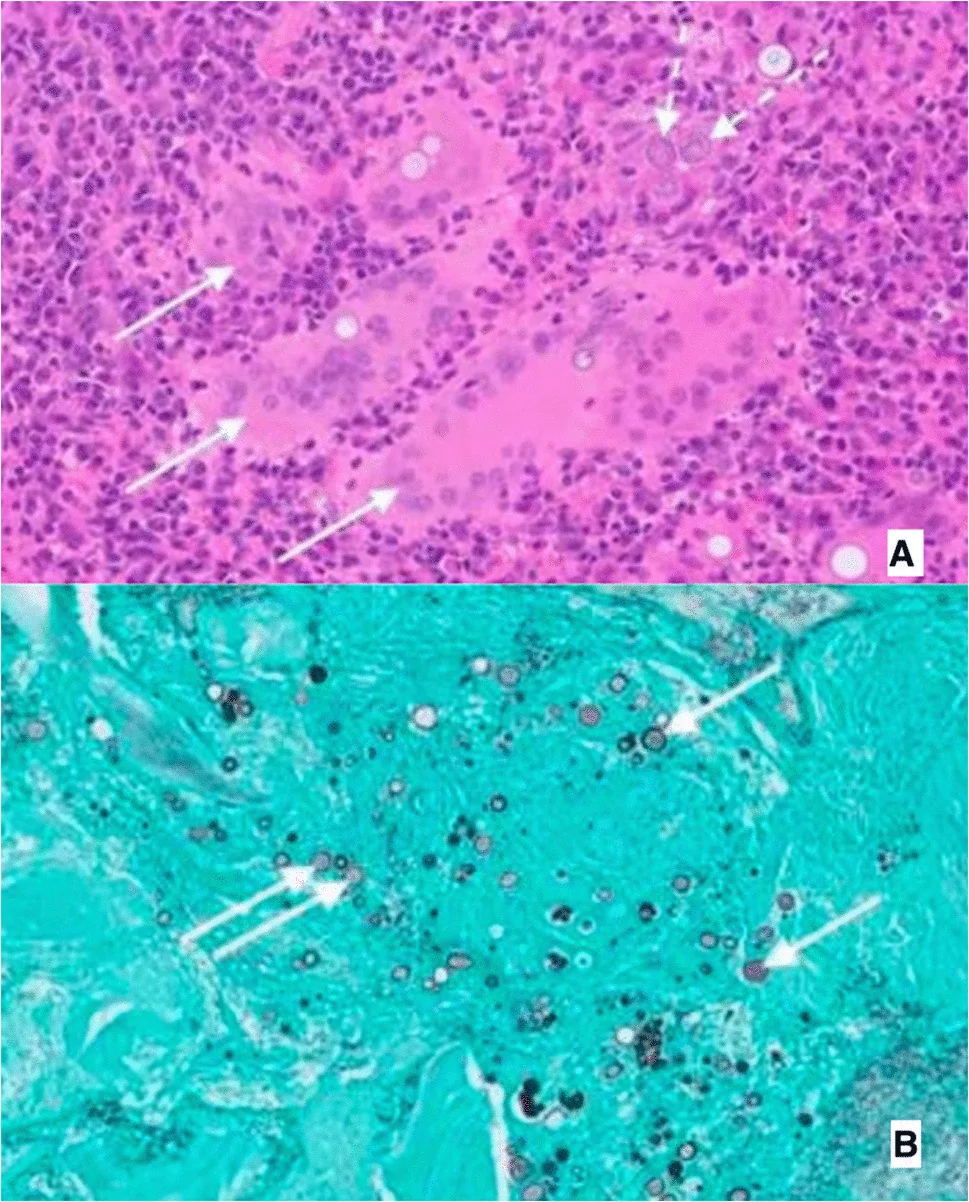
Photomicrograph of a hematoxylin and eosin stained section of the pathological specimen (magnification = 400x) shows numerous multinucleated giant cells (solid arrows) with numerous intracytoplasmic relatively large round fungal organisms (dashed arrows) with surrounding mixed inflammatory infiltrate (**A**). Photomicrograph of the GMS histochemical study performed on the pathological specimen highlights fungal organisms (solid arrows) (**B**)

Leading diagnostic considerations that prompted biopsy included atypical infection versus underlying neoplasm such as Ewing sarcoma or Langerhans cell histiocytosis. CT-guided core needle biopsy was performed under general anesthesia (Fig. 3). Histopathology showed large, thick-walled yeast-like organisms with multiple endospores, granulomatous inflammation, giant cells, mixed acute and chronic inflammatory infiltrates, and bone remodeling (Fig. 4). Gomori methenamine silver stains confirmed fungal spherules (Fig. 4). Bacterial and acid-fast cultures were negative. Fungal culture grew *Coccidioides posadasii/immitis*.

The patient was started on intravenous liposomal amphotericin B at 5 mg/kg/day. After species confirmation, therapy was transitioned to oral itraconazole solution at 10 mg/kg/day divided twice daily, with therapeutic drug monitoring planned to maintain serum trough levels of 1–2 μg/mL. The patient tolerated therapy well without significant adverse events and 6-week imaging follow-up showed response of the lesion to treatment (not shown). At 6-month follow-up, the patient had resumed full academic and athletic activities, reported no residual pelvic pain, and demonstrated preserved hip range of motion. Long-term antifungal therapy is planned for at least three years with serial clinical and imaging monitoring.

## Discussion

Skeletal coccidioidomycosis is an uncommon manifestation of disseminated infection and is particularly rare in children. Its greatest clinical challenge lies in its ability to mimic both bacterial osteomyelitis and primary bone malignancy. Three imaging patterns have been described: focal-lytic lesions, articular involvement, and permeative osseous destruction [8]. In this case, the patient presented without pulmonary symptoms, and imaging demonstrated an aggressive, marrow-replacing lesion of the ilium with osseous lysis, prominent surrounding sclerosis, cortical breach, lamellated periosteal reaction, and an associated heterogeneous soft-tissue component. The combination of MRI findings, including extensive marrow and surrounding soft-tissue edema, areas of rim enhancement, restricted diffusion, representing adjacent inflammatory change with abscess formation, strongly favored an inflammatory or infectious process rather than a primary neoplasm. The absence of matrix mineralization significantly limits the likelihood of the diagnosis of osteosarcoma. Although Ewing sarcoma commonly involves the pelvis and may exhibit bone destruction and periosteal reaction, the degree of surrounding inflammatory change and abscess formation demonstrated in this case would be atypical. Similarly, Langerhans cell histiocytosis may present as a lytic pelvic lesion, although the extensive surrounding edema, necrosis, and abscess formation would be very unusual in this diagnosis. Importantly, the combination of lysis and sclerosis, suggesting chronicity, with marked surrounding inflammatory reaction and abscess formation, favored an indolent infectious or granulomatous process, including atypical organisms such as fungal and mycobacterial pathogens, and prompted biopsy.

Biopsy remains essential for definitive diagnosis, as imaging alone cannot reliably distinguish skeletal fungal infection from malignant or pyogenic processes. CT-guided core needle biopsy in this patient allowed histopathologic identification of yeast-like fungal organisms, granulomatous inflammation, and giant cells, with fungal culture confirming *Coccidioides*. This facilitated timely initiation of antifungal therapy and avoided unnecessary oncologic interventions.

Surgical intervention may be indicated in select cases of skeletal coccidioidomycosis, particularly when there is extensive bone destruction, instability, abscess formation requiring drainage, or failure to respond to medical therapy. In this patient, the lesion was confined to the ilium without joint involvement, sequestrum, or structural compromise, and medical management alone was sufficient. Nevertheless, awareness of the potential need for debridement, drainage, or stabilization is critical in planning comprehensive care for pediatric skeletal fungal infections.

This case highlights several principles directly relevant to clinical practice. Destructive bone lesions in children accompanied by surrounding inflammatory change and abscess formation should prompt consideration of infectious etiologies, including atypical and low-virulence organisms, even in the absence of systemic infectious symptoms. Radiologists must recognize that imaging features of skeletal coccidioidomycosis, including ill-defined lytic destruction with associated sclerosis, periosteal reaction, contiguous soft-tissue inflammatory change, rim-enhancing abscesses, and diffusion restriction, can mimic malignancy but favor infection when considered in aggregate. Early biopsy and culture are essential to confirm the diagnosis and guide appropriate antifungal therapy, thereby preserving musculoskeletal function and preventing long-term morbidity.

## Data Availability

All data analyzed is included in this article.
